# Supplementary material for: Content-rich biological network constructed by mining PubMed abstracts
Source: BMC Bioinformatics. 2004 Oct 8;5:147. doi: 10.1186/1471-2105-5-147 (PMC528731; doi:10.1186/1471-2105-5-147)
Supplement: Additional File 2 — The original results of the above study (non-essential files are deleted to keep the file size under the limit set by BMC bioinformatics). [file 1471-2105-5-147-S2.bz2 › chilibotAdditionalFile2/dip40/256ID8242751E1019/html/CDKN1A_CCNA2.html]

 


 **CDKN1A** and **CCNA2** 
  
Found 163 abstracts in PubMed, retrieved 40.  
 

 What does Google say? 
 PDF only 
| .edu only 

---

**Interactive relationship** (e.g. stimulation, inhibition, etc)

**Inhibitory relationship**- Furthermore, ethanol induced the expression of the cyclin dependent kinase Cdk inhibitor p21 waf1  [ **CDKN1A** ]  cip1  [ **CDKN1A** ] , and inhibited the induction of cyclin A  [ **CCNA2** ] .  Ref: 12079680 Eur J Pharmacol, 2002
- We previously reported that overexpression of HSP25 delayed cell growth, increased the level of p21 waf  [ **CDKN1A** ] , reduced the levels of cyclin D1, cyclin A  [ **CCNA2** ]  and cdc2, and induced radioresistance in L929 cells.  Ref: 11965498 Cell Death Differ, 2002
- They also suggest that induction of p21 cip1  [ **CDKN1A** ]  by the viral protein NS1 arrests cells in G 2 through inhibition of cyclin A  [ **CCNA2** ]  dependent kinase activity.  Ref: 11602746 J Virol, 2001
- p21 Waf1  [ **CDKN1A** ]  Cip1  [ **CDKN1A** ]  exerts a key role in driving this G 2 exit both by inhibiting cyclin B1 Cdk1 and cyclin A  [ **CCNA2** ]  Cdk1 2 complexes, which control G 2 M progression, and by blocking the phosphorylation of pRb family proteins.  Ref: 12881433 EMBO JEMBO J, 2001
- Since cyclin A  [ **CCNA2** ]  exists in the same quaternary complex in the S phase of the cell cycle as the cdk inhibitor p21WAF1 CIP1  [ **CDKN1A** ] , the levels of the two proteins were compared.  Ref: 11745687 J Pathol, 2001
**Stimulatory relationship**- Western blot analysis also shows that DHA greatly reduces the level of cyclin A  [ **CCNA2** ] , while increasing the level of p21 WAF1  [ **CDKN1A** ] , a cellular inhibitor of cyclin A  [ **CCNA2** ]  cyclin dependent kinase 2 activity.  Ref: 12492401 Biochem J< MedlineTA>Biochem J, 2001
- Thus, PD selectively elevates p21 WAF1  [ **CDKN1A** ]  CIP1  [ **CDKN1A** ]  levels and thereby arrests the cell cycle at G1 S phase by down regulating Cyclin A  [ **CCNA2** ]  Cdk2 activity.  Ref: 12819186 Carcinogenesis< MedlineTA>Carcinogenesis, 2001
**Neutral relationship**- Highly potent p21 WAF1  [ **CDKN1A** ]  derived peptide inhibitors of CDK mediated pRb phosphorylation delineation and structural insight into their interactions with cyclin A  [ **CCNA2** ] .  Ref: 12383116 J Pept Res, 2002
- In superficial spreading melanomas, the cyclin A  [ **CCNA2** ]  level was related to tumour thickness, with less expression in thinner lesions p< ; 0.00001, and to Ki 67 p< ; 0.00001 and p21WAF1 CIP1  [ **CDKN1A** ]  p=0.01 scores.  Ref: 11745687 J Pathol, 2001

**Non-interactive relationship** (e.g. studied together, co-existance, homology, etc.)

- METHODS Using cell culture and immunocytochemical techniques, we examined the cell growth, DNA synthesis, expression of PCNA, cyclin A  [ **CCNA2** ] , B 1, D 1, p16 ink4a and p21 cip  [ **CDKN1A** ]  waf1  [ **CDKN1A** ]  of SGC 7901 cells which were treated with various c9, t11 CLA concentrations 2 00 and 200 micromol.L 1 of c 9, t 11 CLA for 24 and 48h, with a negative control 0.1% ethane.  Ref: 11925596 World J Gastroenterol, 2002
- In correlation with its antitumor activity both in vitro and in vivo, Compound 2 induces expression of p21 WAF1  [ **CDKN1A** ]  Cip1  [ **CDKN1A** ] , gelsolin, and keratin 19, while down regulating expression of cyclin A  [ **CCNA2** ]  and cyclin B1 in human cancer cells in a dose dependent manner.  Ref: 12154036 Cancer Res, 2002
- METHODS For each patient, flow cytometry DNA analyses on frozen samples and on immunohistochemical staining were performed, including Ki 67, cyclin A  [ **CCNA2** ] , p53, and p21 waf1  [ **CDKN1A** ]  p21, with assessment of the percentages of positive nuclei were assessed.  Ref: 12627500 Cancer, 2003
- Raf activation increased the expression of cyclin A  [ **CCNA2** ] , cyclin D, cyclin E, and p21 Cip1  [ **CDKN1A** ] , which are associated with G 1 progression.  Ref: 12429936 Cell Cycle< MedlineTA>Cell Cycle, 2003
- The array data enabled us to focus on the role of potential key genes e.g., GADD153, CHK2, p21 WAF1  [ **CDKN1A** ] , cyclin A  [ **CCNA2** ] , CDK1, and DHFR that might be targets of MSA in impeding cell cycle progression.  Ref: 12517777 Cancer Res, 2003
- Expression patterns of cell cycle and or apoptosis associated p27, p21 cip  [ **CDKN1A** ]  waf  [ **CDKN1A** ] , bax, bcl 2, cyclin A  [ **CCNA2** ]  and pro caspase 3 were studied using quantitative Western blotting.  Ref: 11830335 J Hepatol, 2002
- We show that NS1 mediated cell cycle arrest correlates with the accumulation of the cyclin dependent kinase Cdk inhibitor p21 cip1  [ **CDKN1A** ]  associated with both the cyclin A  [ **CCNA2** ]  Cdk and cyclin E Cdk2 complexes but in the absence of accumulation of p53, a potent transcriptional activator of p21 cip1  [ **CDKN1A** ] .  Ref: 11602746 J Virol, 2001
- During G1 phase arrest, the expression level of cyclin D1, cyclin A  [ **CCNA2** ] , and cyclin E was decreased, and the expression of CDK cyclin dependent kinase inhibitor, protein p21 Waf1  [ **CDKN1A** ]  Cip1  [ **CDKN1A** ] , was increased in a time dependent manner in a breast cancer cell MCF 7.  Ref: 12150857 Bioorg Med Chem, 2002
- When cells were synchronized in S phase with aphidicolin and then released, activation of Delta MEKK3 ER\* resulted in the up regulation of p21 CIP1  [ **CDKN1A** ]  and a pronounced inhibition of cyclin A  [ **CCNA2** ]  CDK2 and cyclin B1 CDK1 kinase activity.  Ref: 12444545 Oncogene, 2002
- Panaxadiol selectively inhibits Cyclin A  [ **CCNA2** ]  associated Cdk2 activity by elevating p21WAF1 CIP1  [ **CDKN1A** ]  protein levels in mammalian cells.  Ref: 12819186 Carcinogenesis< MedlineTA>Carcinogenesis, 2002
- CONCLUSION The cell growth and proliferation of SGC 7901 cell is inhibited by c9, t11 CLA via blocking the cell cycle, with reduced expressions of cyclin A  [ **CCNA2** ] , B 1 and D 1 and enhanced expressions of CDKI P16 ink4a and p21 cip  [ **CDKN1A** ]  waf1  [ **CDKN1A** ] .  Ref: 11925596 World J Gastroenterol, 2002
- PS 341 exposure in H460 cells led to stabilization of p53, induction of p21 cip  [ **CDKN1A** ]  waf 1  [ **CDKN1A** ]  and MDM2 expression, an increase in cyclin B and cyclin A  [ **CCNA2** ] , and the activation of cyclin B and cyclin A  [ **CCNA2** ]  kinases.  Ref: 12631620 Clin Cancer Res, 2003
- TSA increased activation of caspase 3 and expression of cyclin A  [ **CCNA2** ] , p2l cip  [ **CDKN1A** ]  waf  [ **CDKN1A** ] , bax and pro caspase 3, while bcl 2 was downregulated.  Ref: 11830335 J Hepatol, 2002
- Similarly, levels of the p21 WAF1  [ **CDKN1A** ]  CIP1  [ **CDKN1A** ]  protein that is associated with the Cyclin A  [ **CCNA2** ]  Cdk2 complex increased, and these increases correlated well with the down regulation of Cyclin A  [ **CCNA2** ]  Cdk2 activity.  Ref: 12819186 Carcinogenesis< MedlineTA>Carcinogenesis, 2002
- However, activation of p42 44 MAPK in the above cells led to the inhibition of DNA synthesis, caused growth arrest, decrease in cyclin A  [ **CCNA2** ]  and upregulation of p21 Cip  [ **CDKN1A** ]  expression in a time dependent manner.  Ref: 12190111 Mol Cell Biochem, 2002
- Immunohistochemical expression of p53, p21 waf1  [ **CDKN1A** ] , rb, p16, cyclin D1, p27, Ki67, cyclin A  [ **CCNA2** ] , cyclin B1, bcl2, bax and bak proteins and apoptotic index in normal thymus.  Ref: 11642719 Histol Histopathol, 2001
